# Evaluating the effectiveness of oral HIV self testing according to distribution models in Cameroon

**DOI:** 10.1038/s41598-024-78444-w

**Published:** 2024-12-28

**Authors:** Yagai Bouba, Audrey Raissa Dzaddi Djomo, Fatima Nkain Mouliom, Adamou Souleymanou, Ebiama Lifanda, Yakouba Liman, Roger Onana, Annie Michele Salla, Lily Claire Ekobika, Gutenberg Tchikangni, Edwige Guissana Omona, Ingrid Kenko Abo’o Myrtho, Ernest Désiré Anaba Mvilongo, Antoine Socpa, Rogers Awoh Ajeh, Marie Jose Essi, Serge Clotaire Billong, Hadja Cherif Hamsatou, Anne Cecile Zoung-Kanyi Bissek

**Affiliations:** 1 UniCamillus - Saint Camillus International University of Health Sciences, Rome, Italy; 2 Central Technical Group, National AIDS Control Committee, Ministry of Public Health, Yaoundé, Cameroon; 3 Division of Health Operational Research, Ministry of Public Health, Yaoundé, Cameroon; 4 University of Yaoundé 1, Yaoundé, Cameroon; 5 Association Camerounaise pour le Marketing Social (ACMS), Yaoundé, Cameroon

**Keywords:** Oral HIV-self testing, Effectiveness, Distribution models, Seropositivity, Linkage to ART, Cameroon, HIV infections, Health policy, Laboratory techniques and procedures

## Abstract

Innovative strategies such as HIV self-testing (HIVST) are useful for identifying hard-to-reach people living with HIV/AIDS (PLHIV), especially in developing settings where considerable gaps still exist in reaching the first 95% UNAIDS target. We evaluated the effectiveness of HIVST in Cameroon using several distribution models and investigated the predictors of HIV seropositivity among self-testers. The study was conducted from 2021 to 2022 in three regions in Cameroon. HIVST kits were distributed according to 5 distribution models: antenatal, postnatal, maternal and child clinics (ANC/PNC/MCH); partners of PLHIV; workplace; community and HIV-testing services (HTS). Overall, 42,687 people received oral HIVST kits, among whom 15.6% were HIV first-testers. Approximately 85% reported on the test outcome; 2.3% (*n* = 825) were reactive, and 75.8% came for test confirmation. After the confirmation test, a concordance of 85% was found with the national algorithm. Overall, the HIV seroprevalence was 1.5% [95% CI: 1.4–1.6]; ANC/PNC/MCHC: 1.9%, partners of PLHIV: 6.9%, workplace: 0.5%, community: 0.1% and HTS: 7.0%, *p* < 0.001. The positivity rate among first-testers was 1.2%. Youths < 25 years had a lower seroprevalence (0.4%) than older people (2.6% and 2.7% for those aged 25–39 and those aged ≥ 40 years, respectively), *p* < 0.001. Seropositivity was negatively associated with secondary distribution, workplace model, community model and age < 25 years. On the other hand, partners of PLHIV model, HTS model, female sex and first-time-testers were positively associated with seropositivity. In Cameroon, oral HIVST is an effective approach for identifying undiagnosed PLHIV, especially when using ANC/PNC/MCHC, partners of PLHIV and HTS distribution models. However, to ensure the successful scale-up of HIVST in Cameroon, guidelines should be revised to fine-tune the target populations for HIVST and optimize the use of resources.

## Introduction

Approximately four decades after its first discovery in 1983, HIV/AIDS remains a major global public health problem. It is worth noting that tremendous advances have been made in combating HIV/AIDS in recent years; however, the recent UNAIDS 2022 HIV/AIDS update report reveals that the global response to HIV/AIDS is now “in danger”^1^. The report also highlights the fact that new HIV infections have increased since 2015 in 38 countries worldwide. The populations most affected are young people aged 15–24, women, girls, and key populations and their partners, who account for up to 70% of these new infections. In Cameroon, according to the results of the Demographic and Health Survey (DHS-IV 2018)^2^, the prevalence of HIV in the general population (15–49 years) is estimated to be 2.7%, and the highest incidence is observed among adolescent girls. Moreover, among key populations, HIV prevalence was 20.6% and 24.3% among men who have sex with men (MSM) and female sex workers (FSWs).

Regarding screening, in Cameroon, as in other countries, certain populations are not easily reached by the usual screening services because of access, stigmatization and criminalization of men who sex with men (MSM) and female sex workers, among the others. This situation was recently exacerbated by the considerable impact of the COVID-19 pandemic, which has considerably influenced the deployment of prevention, screening, and treatment strategies^3–5^, thus increasing the HIV testing gap in Cameroon. To reach the global target for HIV testing in the country, it is therefore imperative to implement new and innovative strategies such as HIV self-testing (HIVST), which has already been proven effective elsewhere^6–8^.

HIV self-testing is a process whereby people collect their own sample (oral fluid or blood), perform their HIV rapid diagnostic test in a private setting, either alone or with a trusted person, and interpret the result. Studies have shown that HIVST can be comparable or even more cost-effective than standard HIV testing^9–11^, especially when implemented by the community. Moreover, although efforts are still needed to improve adherence to testing procedures, the acceptability of HIV treatment by most target populations, such as sex workers^12,13^, MSM^13^, young people^14^, workers^15^, and men^16,17^, is an asset for its implementation. A systematic review and meta-analysis of studies conducted in Africa has also shown that HIVST could contribute to increasing ART uptake^18^, and generally, policy makers seem to favor its adoption^19^. Many therefore believe that HIVST would be the key to achieving testing targets in Africa^19,20^. Consequently, this strategy is now recommended by the World Health Organization (WHO)^21^. However, even if HIVST is an innovative strategy for HIV testing, it faces several challenges in Africa. The most important of these are cultural diversity, inadequate health financing and the integration of HIVST into standard health care services^22–24^. Additionally, challenges exist in confirmatory testing and in measuring linkage to care and prevention services^21,25^.

Recently, Cameroon has adopted a framework for the implementation of HIVST, which targets, among others, key populations and their partners, partners of PLHIV, young women (from 18 years old) in vulnerable situations, partners of HIV + pregnant women and men in vulnerable situations^26^. Given its very recent implementation, data are lacking for efficiently evaluating the overall strategy. To address these challenges, together with other projects under the coordination of the WHO, the Self-Testing Africa Initiative (STAR, https://www.psi.org/project/star/) was put in place to accelerate global access and scale-up of HIV self-testing^27^. STAR supports countries in achieving their global 95–95–95 targets and HIV prevention goals and in creating an enabling environment for the introduction and integration of HIVST based on evidence from several countries^27^. STAR includes the design, implementation and evaluation of a variety of models for the delivery of HIVST and promotes the development of appropriate regulatory and policy environments for HIVST^28^. The STAR Cameroon project aimed to expand the impact of the STAR initiative to increase access to HIVST to remove barriers to the development of the HIV self-testing market. It is important to note that there are only limited studies on HIVST in Cameroon and central Africa in general^13,22^. Therefore, the objective of this study was to evaluate the effectiveness of HIV self-testing in Cameroon.

## Results

### Self-test kit distribution and population characteristics

In the STAR project, a total of 42,687 HIV-ST kits were distributed according to the different distribution models as follows: 10,153 (23.8%) for ANC/MCHC/PNC, 5178 (12.1%) for partners of PLHIV, 10,719 (25.1%) for the workplace, 16,470 (38.6%) for the community and 177 (0.4%) for HTS. Concerning the feedback on the testing outcome, 84.9% (n = 36,230) reported back about the test use. According to the distribution models, the rates of feedback on test use were as follows: ANC/MCHC/PNC (76.4%), PLHIV partner (89.7%), workplace (68.3%), community (99.3%) and HTS (84.9%). Concerning the study population, 64% were from the central region; the majority of them were males (71.5%). Regarding the age structure, 51.3% were < 25 years, 32.5% were 25–39 years, and 16.2% were ≥ 40 years. A proportion of 16.8% declared that they had never been tested for HIV before (Table 1).

**Table 1:** Outcome of HIV testing by a self-tester according to sex, age, region, distribution models and HIV testing history.

Variable	Overall (N = 35,934)	Nonreactive (n = 34,995, 97.4%)	Reactive (n = 825, 2.3%)	Invalid (n = 114, 0.3%)	*P* value
Region, n (%)					
Centre	23,008 (64.0)	22,357 (97.2)	569 (2.5)	82 (0.4)	** < 0.001**
Littoral	5920 (16.5)	5848 (98.8)	69 (1.2)	3 (0.1)	
South	7006 (19.5)	6790 (96.9)	187 (2.7)	29 (0.4)	
Distribution type, n (%)					
Primary	23,854 (66.4)	23,534 (98.7)	218 (0.9)	102 (0.4)	** < 0.001**
Secondary	12,080 (33.6)	11,461 (94.9)	607 (5.0)	12 (0.1)	
Distribution models, n (%)					
ANC/MCHC/PNC	7630 (21.2)	7376 (96.7)	246 (3.2)	8 (0.1)	** < 0.001**
Partners of PLHIV	4539 (12.6)	4157 (91.6)	378 (8.3)	4 (0.1)	
Workplace	7309 (20.3)	7218 (98.8)	85 (1.2)	6 (0.1)	
Community	16,309 (45.4)	16,111 (98.8)	102 (0.6)	96 (0.6)	
HTS	147 (0.4)	133 (90.5)	14 (9.5)	0 (0.0)	
Age categories, years, n (%)					
< 25	18,415 (51.3)	18,142 (98.5)	175 (1.0)	98 (0.5)	** < 0.001**
25–39	11,689 (32.5)	11,252 (96.3)	424 (3.6)	13 (0.1)	
≥ 40	5830 (16.2)	5601 (96.1)	226 (3.9)	3 (0.1)	
Sex, n (%)					
Male	25,710 (71.5)	25,064 (97.5)	570 (2.2)	76 (0.3)	0.144
Female	10,224 (28.5)	9931 (97.1)	255 (2.5)	38 (0.4)	
HIV testing History, n (%)					
At least one	29,703 (82.7)	28,949 (97.5)	679 (2.3)	75 (0.3)	** < 0.001**
Never	6019 (16.7)	5843 (97.1)	137 (2.3)	39 (0.6)	
Unknown	212 (0.6)	203 (95.8)	9 (4.2)	0 (0.0)	

ANC: antenatal clinic, CI: confidence interval, HTS: HIV testing site, MCHC: mother and child health clinic, OR: odds ratio, PLHIV: people living with HIV, PNC: postnatal clinic. *P* values were calculated using the chi-square test.

*P* values underlined in bold are those that are significant, with a statistically significant level fixed at *p* < 0.05.

### HIV self-testing outcomes

Among the 36,230 people, 35,934 (99.2%) had complete information for the analysis. Table 1 shows the outcome of HIV testing by self-tester according to sex, age, region, and distribution model.

The proportions of tests with nonreactive, reactive and invalid results were 97.4% (n = 34,995), 2.3% (n = 825) and 0.3% (n = 114), respectively. According to the distribution type, reactive test results were significantly higher among secondary distribution (5.0%) than for the primary distribution (0.9%), *p* < 0.001. The distribution model with the highest proportion of reactive tests was HTS (9.5%), followed by partners of PLHIV (8.3%) and ANC/MCHC/PNC (3.2%). According to age categories, the highest proportion of reactive tests was observed in those ≥ 40 years (3.9%), followed by those between 25 and 39 years (3.6%). With respect to HIV testing history, we observed a similar proportion of those who had performed HIV testing at least once (2.3%) and those who had never been tested before (2.3%).

It is also important to note that the most invalid tests were observed for the primary distribution type (n = 102, *p* < 0.001), for the community model (n = 92, *p* < 0.001), and for participants < 25 years of age (n = 98, *p* < 0.001) (Table 1).

### Confirmatory test following the national algorithm

A total of 825 and 114 tests were reactive and invalid, respectively; thus required, confirmation at the level of health facilities according to the national algorithm is needed. Only 66.8% (n = 627, among which 625 were reactive tests and 2 were invalid tests) of them underwent a confirmation test. The people who did not perform the test had a significantly higher proportion (n = 112, 98.2%) among those who had an invalid test when compared to those who had a reactive test (n = 200, 24.2%), *p* < 0.001.

According to the distribution type, compared to the primary distribution (43.1%), the secondary distribution showed a greater proportion (79.0%) of those who underwent the confirmation test (*p* < 0.001). Concerning the distribution models, the communities and partners of the PLHIV models had the lowest (24.7%) and highest (84.8%) proportions of those who performed confirmation tests, respectively (*p* < 0.001).

Regarding age groups, compared to older people (25–39 years [78.3%] and ≤ 40 years [76.4%]), those aged less than 25 years accounted for a significantly lower proportion (40.3%) of those who confirmed their test, *p* < 0.001. Finally, regarding the history of HIV testing, compared to those who had previously performed at least one HIVST (69.4%), the first testers represented a lower proportion (54.5%) of those who performed confirmation, *p* < 0.001.

According to the logistic regression model, at the univariate level, region, distribution type, distribution model, HIV testing history and age had a *p* < 0.25.

However, the multivariate model showed that after adjusting for variables that were significant in the univariate model, distribution model, distribution type, age and HIV testing history were significantly associated with performing a confirmatory test. In particular, compared to the ANC/MCHC/PNC model, the partners of the PLHIV and community models had respectively a positive (aOR [95% CI]: 2.357 [2.001–2.778], *p* < 0.001) and negative (aOR [95% CI]: 0.248 [0.154–0.401], *p* < 0.001) associations with performing confirmatory tests, respectively (Table 2). Also, compared to those ≥ 40 years, younger ones had 1.4 times less likely to go for confirmation test.

**Table 2:** Factors associated with performing a confirmation test among HIV self-testers.

Variables	Univariate model		Multivariate model	
	OR (95% CI)	*P*-value	aOR (95% CI)	*P*-value
Regions				
Centre	1		1	
Littoral	1.457 (1.060–2.003)	**0.020**	1.364 (0.927–2.008	0.115
Sud	1.881 (1.042–3.395)	**0.036**	0.911 (0.772–1.076)	0.275
Distribution types				
Primary	1		1	
Secondary	0.202 (0.150–0.270)	** < 0.001**	0.484 (0.325–0.722)	** < 0.001**
Distribution models				
ANC/PNC/MCHC	1		1	
Partners of PLHIV	1.011 (0.307–3.328)	0.985	2.357 (2.001–2.778)	** < 0.001**
Workplace	2.234 (0.678–7.365)	0.186	0.187 (0.110–0.316)	** < 0.001**
Community	0.855 (0.247–2.957)	0.805	0.248 (0.154–0.401)	** < 0.001**
HTS	0.132 (0.039–0.438)	**0.001**	1.866 (0.980–3.556)	0.058
Age categories in years				
< 25	0.208 (0.141–0.307)	** < 0.001**	0.748 (0.566–0.988)	**0.041**
25–39	1.111 (0.759–1.625)	0.588	1.046 (0.887–1.233)	0.595
≥ 40	1		1	
Sex				
Male	1			
Female	1.015 (0.757–1.360)	0.923		
HIV testing history				
At least one	1		1	
Never	0.283 (0.035–2.276)	0.235	1.300 (1.086–1.556)	**0.004**
Unknown	0.150 (0.018–1.225)	0.077	1.535 (0.777–3.031)	0.217

ANC: antenatal clinic, CI: confidence interval, HTS: HIV testing site; MCHC: mother and child health clinic, OR: odds ratio, PLHIV: people living with HIV, PNC: postnatal clinic. All factors with a *p* value < 0.05 were adjusted for in the multivariate analysis. The model was adjusted for region, distribution type, distribution models and age categories.

*P* values underlined in bold are those that are significant. A value of *p* < 0.05 was considered to indicate statistical significance.

### Concordance between HIV reactive tests and the confirmatory test

Of the 625 reactive tests, approximately 85% (n = 532) were confirmed to be reactive with the first test (Determine HIV-1/2). Table 3 shows the distribution of discordant tests according to different variables. In particular, a discordance rate of 33.8% was observed in the primary distribution, compared to only 9.6% in the secondary distribution (*P* < 0.001). According to the distribution models, the community model had the highest percentage (50.0%), followed by the workplace model (36.1%) and the ANC/MCHC/PNC model (19.2%). By age, the percentage of younger patients (< 25 years) was 32.1%, compared to only 8.6% among those aged > 40 years (*P* < 0.001).

**Table 3:** Factors associated with concordance between HIV self-testers and confirmatory tests performed at health facilities.

Variables	Concordance between HIVST & Confirmation test			Univariate model		Multivariate Model	
	Overall N = 625	NO (N = 93, 14.9%)	YES (N = 532, 85.1%)	OR (95% CI)	*P*-value	aOR (95% CI)	*P*-value
Region of distribution, n (%)							
Centre	445	55 (12.4)	390 (87.6)	1		1	
Littoral	52	21 (40.4)	31 (59.6)	0.208 (0.112–0.388)	** < 0.001**	0.313 (0.090–1.090)	0.068
Sud	128	17 (13.3)	111 (86.7)	0.921 (0.514–1.650)	0.782	1.715 (0.869–3.384)	0.120
Distribution type, n (%)							
Primary	136	46 (33.8)	90 (66.2)	1		1	
Secondary	489	47 (9.6)	442 (90.4)	0.208 (0.131–0.331)	** < 0.001**	1.095 (0.213–5.645)	0.913
Distribution models, n (%)							
ANC/PNC/MCHC	182	35 (19.2)	147 (80.8)	1		1	
Partners of PLHIV	324	12 (3.7)	312 (96.3)	6.190 (3.123–12.272)	** < 0.001**	7.421 (3.652–15.079)	** < 0.001**
Workplace	61	22 (36.1)	39 (63.9)	0.422 (0.223–0.800)	**0.008**	1.037 (0.143–7.507)	0.971
Community	48	24 (50.0)	24 (50.0)	0.238 (0.121–0.468)	** < 0.001**	0.462 (0.065–3.304)	0.442
HTS	10	0 (0.0)	10 (100)	384,636,867	0.999	466,343,719	0.999
Age categories, n (%)							
< 25	109	35 (32.1)	74 (67.9)	0.199 (0.103–0.388)	** < 0.001**	0.277 (0.104–0.738)	**0.010**
25–39	342	43 (12.6)	299 (87.4)	0.656 (0.353–1.218)	0.181	0.429 (0.260–0.855)	**0.016**
≥ 40	174	15 (8.6)	159 (91.4)	1		1	
Sex, n (%)							
Male	430	68 (15.8)	362 (84.2)	1			
Female	195	25 (12.8)	170 (87.2)	0.783 (0.478–1.182)	0.331		
HIV testing history, n (%)							
At least one	521	70 (13.4)	451 (86.4)	1		1	
Never	96	22 (22.9)	74 (77.1)	0.522 (0.305–0.894)	**0.018**	0.614 (0.321–1.173)	0.140
Unknown	8	1 (22.9)	7 (87.5)	1.086 (0.132–8.965)	0.939	1.593 (0.138–18.359)	0.709

This refers to the disagreement between the results of the HIVST kit (reactive) and the determination of HIV-1/2 (nonreactive), which is the first test in the national algorithm.

ANC: antenatal clinic, CI: confidence interval, HTS: HIV testing site; MCHC: mother and child health clinic, OR: odds ratio, PLHIV: people living with HIV, PNC: postnatal clinic. All factors with a *p* value < 0.05 were adjusted for in the multivariate analysis. The model was adjusted for region, distribution type, distribution models, age categories and HIV testing history.

*P* values underlined in bold are those that are significant. A value of *p* < 0.05 was considered to indicate statistical significance.

According to the regression model, in the univariate analysis, region, distribution type, distribution model, age and HIV testing history were significantly associated with discordant results. However, in the multivariate model, after adjusting for variables that were significant in the univariate model, only the distribution model and age remained significantly associated. We observed that, compared to the ANC/MCHC/PNC model, the partners of the PLHIV model were significantly and positively associated with good agreement (aOR [95% CI]: 7.421 [3.652–15.079]). Moreover, compared to those aged ≥ 40 years, those aged 25–39 (aOR [95% CI]: 0.429 [0.260–0.855]) and < 25 years (aOR [95% CI]: 0.277 [0.104–0.738]) were negatively and significantly associated with having discordant results between self-testers and confirmation tests at health facilities.

### HIV seropositivity among self-testers

The overall HIV seroprevalence after confirmation following the national algorithm was 1.5%, with a higher prevalence in the Centre (1.7%) and South (1.6%) regions than in the Littoral region (0.5%), *P* < 0.001. The secondary distribution (3.7%) had a greater prevalence than did the primary distribution (0.4%), *P* < 0.001.

According to age, compared to individuals 25–39 years (2.6%) and those ≥ 40 years (2.8%), we found that younger individuals < 25 years had a significantly lower seroprevalence (0.4%), *p* < 0.001. Looking at the seroprevalence according to HIV testing history, only a slight difference was found between individuals who reported having been tested at least once (1.5%) and first testers (1.2%), *p* < 0.104.

After performing the univariate analyses, all the variables (Table 4) were found to be a potential confounder (*p* < 0.25). After adjusting for all these variables in the multivariate model, we found that all of them were independently associated with HIV seropositivity (Table 4). In particular, for the distribution models, the workplace and community models were negatively and independently associated with HIV seropositivity, while the partners of PLHIV and HTS models were positively and independently associated with HIV seropositivity. Compared to those of the ANC/PNC/MCHC model, the partners of the PLHIV and HTS models were approximately 4 times and 3 times more likely to be HIV positive, respectively, among HIV self-testers. Concerning HIV testing history, compared to those who had tested at least once, those who had never been tested for HIV had significantly greater odds of being HIV positive (Table 4).

**Table 4:** Factors associated with HIV seropositivity among oral HIV self-testers.

Variables	HIV status			Univariate model		Multivariate Model	
	Overall N = 35,620	Negative N = 35,089, 98.5%	Positive N = 531, 1.5%	OR (95% CI)	*P*-value	aOR (95% CI)	*P*-value
Region, n (%)							
Centre	22,802	22,412 (98.3)	390 (1.7)	1		1	
Littoral	5901	5870 (99.5)	31 (0.5)	0.303 (0.210–0.438)	< 0.001	0.525 (0.285–0.968)	**0.039**
Sud	6917	6807 (98.4)	110 (1.6)	0.929 (0.750–1.149)	0.496	0.682 (0.542–0.858)	**0.001**
Distribution type, n (%)							
Primary	23,671	23,581 (99.6)	90 (0.4)	1		1	
Secondary	11,949	11,508 (96.3)	441 (3.7)	10.041 (7.995–12.609)	< 0.001	0.366 (0.236–0.567)	** < 0.001**
Distribution models, n (%)							
ANC/PNC/MCHC	7558	7411 (98.1)	147 (1.9)	1		1	
Partners of PLHIV	4480	4169 (93.1)	311 (6.9)	3.761 (3.080–4.593)	< 0.001	3.432 (2.741–4.298)	** < 0.001**
Workplace	7280	7241 (99.5)	39 (0.5)	0.272 (0.190–0.387)	< 0.001	0.153 (0.077–0.305)	** < 0.001**
Community	16,159	16,135 (99.9)	24 (0.1)	0.075 (0.049–0.116)	< 0.001	0.031 (0.016–0.060)	** < 0.001**
HTS	143	133 (93)	10 (7)	3.791 (1.953–7.357)	< 0.001	2.588 (1.290–5.190)	**0.007**
Age categories, n (%)							
< 25	18,250	18,177 (99.6)	73 (0.4)	0.142 (0.107–0.187)	< 0.001	0.594 (0.422–0.836)	**0.003**
25–39	11,594	11,295 (97.4)	299 (2.6)	0.935 (0.769–1.137)	0.501	0.855 (0.699–1.047)	0.130
≥ 40	5776	5617 (97.2)	159 (2.8)	1		1	
Sex, n (%)							
Male	25,494	25,133 (98.6)	361 (1.4)	1		1	
Female	10,126	9956 (98.3)	170 (1.7)	1.189 (0.989–1.429)	0.065	1.478 (1.191–1.835)	** < 0.001**
HIV testing history, n (%)							
At least one	29,470	29,020 (98.5)	450 (1.5)	1		1	
Never	5939	5865 (98.8)	74 (1.2)	0.814 (0.635–1.042)	0.102	1.763 (1.357–2.289)	** < 0.001**
Unknown	211	204 (96.7)	7 (3.3)	2.213 (1.036–4.728)	0.040	2.599 (1.179–5.726)	**0.018**

ANC: antenatal clinic, CI: confidence interval, HTS: HIV testing site; MCHC: mother and child health clinic, OR: odds ratio, PLHIV: people living with HIV, PNC: postnatal clinic. All factors with a *p* value < 0.05 were adjusted for in the multivariate analysis. The model was adjusted for region, distribution type, distribution models, age categories and HIV testing history.

*P* values underlined in bold are those that are significant. A value of *p* < 0.05 was considered to indicate statistical significance.

## Discussions

HIVST is a new strategy for reaching hard-to-test populations, but its integration into the national strategy in Cameroon is recent. Although previous studies have demonstrated the acceptability and feasibility of HIVST in some populations in Cameroon and in similar settings^12–14,16,17^, more studies are needed to refine the strategy and the models used. The STAR Cameroon project was therefore implemented to ensure the successful scaling up of this new approach, which could be vital for achieving the first 95% of UNAIDS targets by 2025[25]. Another important point in the successful implementation of HIVST in countries where access to HIV services seems limited for hard-to-reach populations is enabling the environment and creating demand, especially in multicultural contexts such as Cameroon^22–24^. In this study, a good number of HISVST kits were successfully distributed, with approximately 85% of individuals providing feedback on the test outcomes. This high performance is justified by the fact that, in the course of the project, there was a close follow-up after picking up the test kits, especially in the community model. To gain from the advantages offered by HIVST^20^, addressing cultural diversity and increasing efforts in the uptake and acceptability of HIV self-testing services in routine practice are of paramount importance^20,26^.

Overall, reactive oral HIVST represented only 2.3% (lower than the national HIV prevalence, which is 2.7%), which seems lower than that reported in other studies. This can be explained by the fact that, in general, other studies focused on populations with much higher HIV risk, such as MSM and female sex workers^29^. Of note, in the case of this study, more than 50% of the 42,687 kits were distributed through community and workplace models, which represented a low positivity rate (0.6% and 1.2%, respectively). Interestingly, only a few invalid tests were observed, compared to up to 15% in other studies^30^; suggested that most of them did not experience major difficulties in performing the test^13^. Most of the invalid tests (84.2%) were observed in the community distribution model. The people most targeted by this model are youths (during social and sports events), and up to 86% of the invalids were reported among those < 25 years of age. This suggests that even though a preference for HIVST was previously shown among youth^14^, this model needs to be adjusted; the environment must be suitable, and tests must be performed in private settings. Another aspect that should not be considered is the continuous improvement of rapid diagnostic tests for self-testing, particularly to make sample collection easier and to simplify instructions for use^31^.

Although some studies found greater uptake and frequency of HIV testing associated with HIVST than with standard HTS^32^, challenges remain in confirming the tests using the national algorithm. In this study, only approximately 67% of people with reactive or invalid results were confirmed. This proportion was especially worrisome among those who had an invalid test result. Our results demonstrate that the model of partners of PLHIV shows great promise, while test confirmation in the community model is low (Table 2). A study in Kenya reported that linkage to confirmatory tests was 50% among partners of pregnant and postpartum women^33^. Other studies reported that men in particular struggle to follow up with confirmatory tests because of the social perceptions of masculine roles^16^. Stakeholders in the implementation of HIVST must therefore set up an efficient system to limit the number of people lost to follow-up after the first test. Additional studies are also required to deepen the understanding of why this happens. For example, the integration of verification technologies might be needed in real time, and patients might need to be connected to appropriate post-HIVST services^34^.

The agreement between the oral HIVST kits and the national algorithm was approximately 85% for the reactive tests (Table 3). The two factors that were independently associated with agreement were the distribution model and age of the participants. In particular, there were approximately 7 times greater odds of having concordant results for partners of PLHIV than for those in the ANC/PNC/MCHC model. Notably, the concordance rate in the community model was only 50%, while 100% concordance was observed in the HTS model. This observation suggests that, in the Cameroonian setting, among the models under consideration in this study, the partners of the PLHIV has the greatest potential. Overall, sensitivity was less than that reported in a randomized implementation trial in Uganda (90%)^35^. Even though the study concluded that unsupervised HIVST is feasible in rural Africa and may be noninferior to provider supervised HIVST, we believe that using supervised testing might significantly improve the performance observed in some models in our study. On the other hand, a systematic review revealed that the agreement between trained health care workers and self-testers ranges from 85.4 to 100%^31^. Therefore, HIVST seems to be reliable and can be used to accurately perform rapid HIV diagnostic tests. However, particular attention should be given to improving the performance among youths and in some models.

Concerning seroprevalence, according to our study, the prevalence of HIV in the population of self-testers was only 1.5% (in contrast to 2.7% for the national prevalence), with less than 1% in the workplace and community models. In view of the scale-up of HIVST, especially the use of community and workplace models, it is imperative to adjust the strategy. Although the distribution models did not target key populations for which the prevalence is generally high according to HIVST^29^, the prevalence rates of partners of the PLHIV and HTS models were high, at 6.9% and 7.0%, respectively (Table 4). A study among adult populations in Kenya reported an HIV prevalence of 14.6% among self-testers^30^, which is much greater than our results. This difference is probably attributed to differences in sample size, population characteristics and country epidemiological profiles. The increased seroprevalence among partners of PLHIV might demonstrate an increase in self-reported partner HIV testing when HIVST is available^16^. Other studies have suggested, for example, that distributing HIV-positive women HIVST kits to their male partners is feasible and safe and that providers who have challenges reaching male partners with testing could consider HIVST^36^. The regression analysis showed that the models of partners of PLHIV and HTS, the female sex, and those who had never been tested before were positively and independently associated with being HIV positive (Table 4). Beyond testing, generally, the proportion of people linked to ART following HIVST is comparable to that of standard facility-based HTS^37^. However, studies have advocated the importance of developing effective approaches to maximize linkage to care among those testing positive through HIVST, especially among men^16^. The entire process, including the use of test kits, confirmation tests and linkages to the ART, should be addressed by using accurate and appropriate measurement strategies^21^.

The findings of this study support the usefulness of the HIVST in the Cameroonian context. It should be noted that several barriers that impedes its implementation must be addressed. Similarly, to other African settings, Cameroon has a high cultural diversity. Given that the self-testing strategy is also designed to reach those with low access to HIV testing (e.g., people in remote areas), efforts should be done to alleviate cultural barriers, including the provision of services and testing instructions in local languages^22^. Moreover, the health system should take advantage of the ongoing task shifting process from medical to non-medical trained persons and the HIV differentiated service delivery, to integrate and make HIVST strategy sustainable. If these challenges, together with the financing gaps are resolved, HIVST would potentially contribute to reducing the testing gaps and help to track unidentified PLHIV.

This study has several limitations. First, in the analysis, the data collected through ANC, MCH and PNC could not be analyzed separately, because the data collection tool did not initially individuate each of these points of entry. Second, the project did not collect data on some key risk behavior and sociodemographic factors that could have enriched this work. In fact, the availability of data on sociodemographic characteristics such as education level, income, and sexual behaviour among the others could have provided a more comprehensive understanding of the factors influencing the use of HIVST. More research is needed to overcome these limitations, characterize the best HIVST delivery models, and to ensure linkage to- and success of- ART.

## Conclusions

In Cameroon, evidence from the STAR Initiative showed that oral HIV self-testing is an effective approach for identifying undiagnosed PLHIV, with a good feedback rate on test kit use (85%), a moderate test confirmation rate (67%) and good concordance between self-testers and trained health practitioners (85%). This approach was able to detect several HIV-infected individuals, especially using ANC/PNC/MCHC, partners of PLHIV and HTS distribution models. However, to ensure the successful scale-up of HIVST in Cameroon, guidelines should be revised to fine-tune the target populations, especially in community and workplace distribution models, to optimize the use of limited resources.

## Method

### Study area and population

This study was conducted in Cameroon, a culturally diverse country in Central Africa with a population of approximately 27 800 000 inhabitants. Cameroon is divided into ten regions, but the STAR project was conducted in three regions: the littoral (economic capital), centre (political capital), and southern regions. A total of 96 health facilities were involved in the project. Regarding the companies that were included, those in the Littoral region, which has a large workforce, were mainly targeted. The target population of the STAR Cameroon project included partners of pregnant women living with HIV, partners of PLHIV, young people (18–24 years) in vulnerable situations and employees of large companies.

### Type of study

We conducted a cross-sectional and analytic study. The study was conducted from April 2021 to August 2022 in the three regions selected for the implementation of the project.

### Distribution types and models

HIV test kits were distributed in two different ways: directly to the people involved (primary distribution) or through a third party (secondary distribution). The secondary distribution was especially used in the context of index case testing and screening of vulnerable/high risk individuals (PLHIV and person at high risk of HIV collect the test for their partner(s)). As such, this method was especially used in the health facility-based methods (ANC/MCHC/PNC, PLHIV and HTS). Three main distribution models were used: community-based distribution (implemented between October 2021 and December 2021), workplace distribution (implemented between July 2021 and April 2022), and health facility-based distribution (implemented between April 2021 and April 2022). Concerning the community-based distribution model, four modes of distribution were used: targeted community distribution, hotspot distribution, door-to-door distribution, and community-led distribution. The health facility-based distribution included ANC/MCHC/PNC (antenatal clinic, maternal and child health clinic and postnatal clinic), partners of PLHIV receiving antiretroviral treatment (excluding HIV + pregnant women), and distribution at voluntary counseling and testing sites. Finally, workplace distribution was determined through the hygiene and health committees of the companies, which first received appropriate training. Concerning the workplace model, test kits were proposed to workers at their workplace by trained personnel. Concerning the health facility-based model, HIV self-test kits were proposed to PLHIV at the treatment centers and to people in other entry points such as antenatal clinic. The distribution models were selected to reach some of the specific target populations of the national HIVST framework. The community model aimed at reaching vulnerable women in hotspots and youths, the workplace model to reach men and women, and the health facility-based model to reach partners of PLHIV and people at risk of HIV.

### Sample types and screening algorithm

The test kit used for HIVST was Oraquick HIV-1/2 (OraSure Technologies Inc., Thailand). Regarding the type of sample, only oral fluid was used by the HIV self-testers. Instructions on how to perform and interpret the test were given to all self-testers. In the case of a reactive or invalid result, a confirmatory test was performed in a health facility following the national HIV testing algorithm. The national HIV screening algorithm in Cameroon includes the first test Determine HIV-1/2 (Abbott diagnostics, USA) and the second test HIV-1/2 colloidal gold (Oraquick HIV-1/2 [OraSure Technologies Inc., Thailand], Hexagon HIV-1/2 [Human Biochemica, Germany] or KHB HIV-1/2 [Shanghai Kehua Bioengineering Co., Ltd, China]). The confirmatory tests were carried out by trained health care workers at the participating health facilities using a serial testing algorithm and were exclusively performed using blood samples (whole blood, plasma or serum).

### Interpretation of the tests

The tests were interpreted following the manufacturers’ instructions as follows:

(i) The test was considered “invalid” when the “control” band was absent, regardless of the presence of a band in the “test” area. (ii) The test was considered “nonreactive” when the “control” band was present but absent in the “test” area. (iii) The test was considered ‘reactive’ when both the ‘control’ and ‘test’ bands were present, regardless of band intensity.

With regard to the participants’ serological status, the results were interpreted as follows:

(i) The serological status was considered “negative” when the first test was “nonreactive”. In this case, there is no need to proceed to the second test. (ii) The serological status was considered “positive” when both the first and second tests were “reactive”. (iii) 'The serological status was considered “Inconclusive” when the first test was ‘reactive’ but the second test was ‘nonreactive’.

### Data collection and quality assurance

The companies’ focal points and community mobilization agents were responsible for collecting and entering individual data into the national database (DHIS2). Data compilation and analysis were performed via DHIS2 by the Cameroon Association for Social Marketing (ACMS). The first sheets of the compiled HIVST register were systematically removed after each campaign, and a second sheet was used to complete the follow-up data, which was later sent to the quality assurance officer in charge of entering these data into the DHIS2 database. To ensure quality, all the data were reviewed and validated during meetings. All participants with incomplete data regarding the main variables were excluded from our analysis.

### Data analysis

After the data were cleaned in a Microsoft Excel spreadsheet, the file was imported into SPSS version 26 (SPSS Inc., Chicago, IL, USA). The data are summarized as number and proportions in the tables. The categorical variables of interest were compared among the groups using the Chi-square test or Fisher’s exact test, as appropriate. A binary logistic regression model was used to identify factors independently associated with the dependent variables of interest among self-testers. All the variables with *p*-values < 0.25 in the univariate analysis were fitted and adjusted for in the multivariate analysis. The statistical significance was set at *p* < 0.05.

### Ethical considerations

This study was conducted in accordance with the principles of the Declaration of Helsinki and national regulations. HIV self-testing is one of the testing strategies that was included in the national strategic plan for the fight against HIV/AIDS in Cameroon. To operationalize this strategy, a national framework for the implementation of HIV self-testing was adopted by the Ministry of Public Health. The STAR project in Cameroon helped the country operationalize the use of self-testing among the target populations described in the national framework of HIV self-testing. Informed consent was obtained from all the participants included in this study. The study protocol was approved by the Institutional Ethical Review Board of the Faculty of Medicine and Biomedical Sciences (FMBS) of the University of Yaoundé 1 (Reference No. 0024/UY1/FMSB/VDRC/CSD) on September 13, 2021. However, all the required administrative authorizations at both the national (Central Technical Group of the National AIDS Control Committee) and regional levels (Regional delegation for public health and Regional Technical Group—National AIDS Control Committee) were obtained. However, given that personal data were collected, confidentiality was maintained by using only codes to identify participants. In fact, all the information was entered into the DHIS2 system, which is a national information system in which only authorized persons have access.

### Conference presentation

The results of this manuscript were in part presented as a poster (Abstract No 928) at the Conference on Retroviruses and Opportunistic Infections 2023 in Seattle, Washington, on February 19 to 22, 2023.

## Data Availability

The dataset used during the current study are available from the corresponding author on reasonable request.
